# Stress Mediating Genes in Aging, Health, and Longevity Traits: Effects of Multiple Interactions

**DOI:** 10.1093/geroni/igaa057.915

**Published:** 2020-12-16

**Authors:** Anatoliy Yashin, Dequing Wu, Konstantin Arbeev, Arseniy Yashkin, Galina Gorbunova, Igor Akushevich, Matt Duan, Svetlana Ukraintseva

**Affiliations:** 1 Duke University, Durham, North Carolina, United States; 2 Duke University, Durham, United States; 3 Duke.University, Durham, North Carolina, United States

## Abstract

Persistent stress of external or internal origin accelerates aging, increases risk of aging related health disorders, and shortens lifespan. Stressors activate stress response genes, and their products collectively influence traits. The variability of stressors and responses to them contribute to trait heterogeneity, which may cause the failure of clinical trials for drug candidates. The objectives of this paper are: to address the heterogeneity issue; to evaluate collective interaction effects of genetic factors on Alzheimer’s disease (AD) and longevity using HRS data; to identify differences and similarities in patterns of genetic interactions within two genders; and to compare AD related genetic interaction patterns in HRS and LOADFS data. To reach these objectives we: selected candidate genes from stress related pathways affecting AD/longevity; implemented logistic regression model with interaction term to evaluate effects of SNP-pairs on these traits for males and females; constructed the novel interaction polygenic risk scores for SNPs, which showed strong interaction potential, and evaluated effects of these scores on AD/longevity; and compared patterns of genetic interactions within the two genders and within two datasets. We found there were many genes involved in highly significant interactions that were the same and that were different within the two genders. The effects of interaction polygenic risk scores on AD were strong and highly statistically significant. These conclusions were confirmed in analyses of interaction effects on longevity trait using HRS data. Comparison of HRS to LOADFS data showed that many genes had strong interaction effects on AD in both data sets.

